# Choroid plexus LAT2 and SNAT3 as partners in CSF amino acid homeostasis maintenance

**DOI:** 10.1186/s12987-020-0178-x

**Published:** 2020-02-11

**Authors:** Elena Dolgodilina, Simone M. Camargo, Eva Roth, Brigitte Herzog, Virginia Nunes, Manuel Palacín, Francois Verrey

**Affiliations:** 1 Institute of Physiology, University of Zurich, Winterthurerstrasse 190, 8057 Zurich, Switzerland; 2 Genes, Disease and Therapy Program, Molecular Genetics Laboratory-IDIBELL, Barcelona, Spain; 3 U730 and U731, Centro de investigacion Biomédica en Red de Enfermedades Raras, Barcelona, Spain; 4 Genetics Section, Department of Physiological Sciences, Faculty of Medicine and Health Sciences, University of Barcelona, Barcelona, Spain; 5 Institute for Research in Biomedicine, The Barcelona Institute of Science and Technology, Barcelona, Spain; 6 Department of Bioquimica I Biomedicina Molecular, Facultat de Biología, Universitat de Barcelona, Barcelona, Spain; 7 Swiss National Centre of Competence in Research Kidney.CH, University of Zurich, Zurich, Switzerland

**Keywords:** Blood–cerebrospinal fluid barrier, CSF, Amino acid transporters, Localization, Homeostasis

## Abstract

**Background:**

Cerebrospinal fluid (CSF) is mainly produced by the choroid plexus (CP) located in brain ventricles. Although derived from blood plasma, it is nearly protein-free (~ 250-fold less) and contains about 2–20-fold less free amino acids, with the exception of glutamine (Gln) which is nearly equal. The aim of this study was to determine which amino acid transporters are expressed in mouse CP epithelium in order to gain understanding about how this barrier maintains the observed amino acid concentration gradient.

**Methods:**

Expression of amino acid transporters was assessed in isolated choroid plexuses (CPs) by qRT-PCR followed by localization studies using immunofluorescence with specific antibodies. The impact of LAT2 (*Slc7a8*) antiporter deletion on CSF amino acids was determined.

**Results:**

The purity of isolated choroid plexuses was tested on the mRNA level using specific markers, in particular transthyretin (Ttr) that was enriched 330-fold in CP compared to cerebral tissue. In a first experimental round, 14 out of 32 *Slc* amino acid transporters tested on the mRNA level by qPCR were selected for further investigation. Out of these, five were considered highly expressed, SNAT1 (*Slc38a1*), SNAT3 (*Slc38a3*), LAT2 (*Slc7a8*), ASC1 (*Slc7a10*) and SIT1 (*Slc6a20b*). Three of them were visualized by immunofluorescence: SNAT1 (*Slc38a1*), a neutral amino acid-Na^+^ symporter, found at the blood side basolateral membrane of CP epithelium, while SNAT3 (*Slc38a3*), an amino acid-Na^+^ symporter and H^+^ antiporter, as well as LAT2 (*Slc7a8*), a neutral amino acid antiporter, were localized at the CSF-facing luminal membrane. In a LAT2 knock-out mouse model, CSF Gln was unchanged, whereas other amino acids normally 2–20-fold lower than in plasma, were increased, in particular the LAT2 uptake substrates leucine (Leu), valine (Val) and tryptophan (Trp) and some other amino acids such as glutamate (Glu), glycine (Gly) and proline (Pro).

**Conclusion:**

These results suggest that Gln is actively transported by SNAT1 from the blood into CP epithelial cells and then released luminally into CSF via SNAT3 and LAT2. Its efflux via LAT2 may drive the reuptake from the CSF of essential amino acid substrates of this antiporter and thereby participates to maintaining the amino acid gradient between plasma and CSF.

## Background

Extracellular fluid compartments of the CNS include the cerebrospinal fluid (CSF) filling the intracerebral ventricles and the subarachnoid spaces, the brain interstitial fluid (ISF) surrounding the different cells of the brain parenchyma and blood in cerebral blood vessels [1]. In humans around 500–600 mL of CSF is produced in 24 h, the majority of which (~ 80%) is secreted into the brain ventricles by the choroid plexuses (CP), while only a small portion (~ 20%) comes from ISF [2, 3]. Its composition is also influenced by its contact with the blood–arachnoid barrier (BAB). The CSF content of major ions such as Na^+^, Mg^2+^, Cl^−^ and HCO_3_^−^ is generally comparable with that of plasma, but more tightly regulated [3]. Notably, strong concentration gradients between plasma and CSF were reported for proteins (~ 250 fold) and proteinogenic amino acids (AAs) [2–20-fold, with the exception of glutamine (Gln)] in independent studies [3–5]. Amino acids being natural components of extracellular fluids and relatively easily measurable, their concentration levels in CSF, have been proposed over the last 30 years to represent potential diagnostic biomarkers for many neurological conditions such as Alzheimer disease (AD), amyotrophic lateral sclerosis (ALS), motor neuron disease and essential tremor. In the case of ALS data about changes in the level of the major excitatory neurotransmitter glutamate (Glu) remains controversial, while neutral non-essential amino acids as Gln and alanine (Ala) have been reported to be elevated in two independent studies [6–8]. In contrast, CSF Glu concentration was shown to raise slightly during the brain disorder essential tremor, while the levels of other neurotransmitters (aspartate (Asp), GABA) and some amino acids [serine (Ser), threonine (Thr), Gln, glycine (Gly) and ornithine (Orn)] declined [9]. Elevated Glu concentrations were also detected in CSF samples from patients with AD, however no correlation between this increase and clinical features was identified [10, 11]. Observations about CSF levels of other AAs during AD are more contradictory [10–14]. Taken together, these numerous observations confirm that knowledge about the regulatory mechanisms underlying the maintenance of CSF AA homeostasis is important and relevant for clinical practice.

Because brain fluid homeostasis is essential for proper CNS function, it is effectively maintained in adult mammals by both the blood–brain barrier (BBB) and the blood–CSF (BCSF) barriers. As mentioned above, the choroid plexus (CP) is a main component of the BCSF that mediates most CSF production. Its epithelial cells are highly polarized and display distinct basolateral (blood-facing) or luminal (CSF-facing) membrane localizations of their ion, water and solute transport proteins [2]. The CP is thus expected to be the main CSF amino acid influx and homeostasis site, in particular in view of the substantially lower amino acid concentration in brain ISF and the fact that the BAB is not considered as a crucial entry pathway but rather as a site of waste and drug clearance [15–17].

Consequently, to understand how CSF AA levels are controlled, it is essential to know not only which amino acid transporters (AATs) are expressed in CP, but also their membrane localization. So far, mRNAs of a number of amino acid transporters were identified in CP epithelium by microarray analysis or in situ hybridization, in particular of the imino acid-Na^+^ symporter SIT1 (*Slc6a20*) and the small neutral non-essential amino acid-Na^+^ symporter/H^+^ antiporter SNAT3 (*Slc38a3*) and, at a lower level, the two antiporters LAT2 (large neutral amino acid transporter 2) (*Slc7a8*) and y^+^LAT2 (*Slc7a6*) [18–21]. The use of different approaches in different studies however limits the possibility to compare this information. Subcellular localization data has to our knowledge been published as yet for two amino acid transporters, specifically for SNAT3 of which the luminal localization has been inferred from functional experiments and for EAAT3 (Slc1a1) that was localized also to the luminal CP epithelium membrane using immunofluorescence [22, 23].

The aim of the present study was to identify AATs, which play key roles in maintaining homeostatic AA concentrations in CSF. To achieve this goal, we did a detailed comparative analysis of 14 AATs expressed in the CP using qRT-PCR and subsequently localized the three most abundantly expressed transporters by immunofluorescence. Finally, we investigated the consequences of the knockout (KO) of *Lat2* (*Slc7a8*^−*/*−^) on AA levels in CSF and based on our result suggest a possible transport schema supporting CSF AA concentration homeostasis.

## Materials and methods

### Animals

Male and female 8- to 16 weeks old wild type and LAT2 (*Slc7a8*) KO (knockout) [24] C57BL/6J mice were used (Charles River (Crl), Germany and in-house breeding). Animals were kept in standard cages under 12-h light/dark cycle (7:00 h/19:00 h) with free access to food and tap water. All animal experiments were conducted in accordance with the Swiss federal and cantonal law and performed with the approval of the Swiss Veterinary Council, Approval number 205/2016.

### CSF and terminal blood collection

CSF samples were obtained as previously described [25]. After CSF collection blood was carefully withdrawn by cardiac puncture, transferred to an Eppendorf tube with heparin and kept on ice. As soon as the last sample was obtained all blood samples were centrifuged for 10 min at 10,000*g* (4 °C) to separate plasma.

### Immunofluorescence

Deeply anaesthetized mice were transcardially perfused with ice-cold PBS (pH 7.4), brains were removed, cut into two halves and fixed in 4% PFA at 4 °C overnight. The next day the right half of each brain was washed in PBS, incubated in 30% sucrose and afterwards frozen in OCT embedding matrix (CellPath Ltd, Newtown, UK) on dry ice. The left half of each brain was washed in PBS, stepwise incubated in ethanol of 20%, 40% and 60%, stored in 70% till paraffinization on Microm spin tissue processor STP-120 (Microm International GmbH, part of Thermo Fischer Scientific, Walldorf, Germany) and subsequently embedded in paraffin. Saggital 10 µm thick cryosections were cut on a cryostat (Leica CM1850, Leica Biosystems Nussloch GmbH, Nussloch, Germany) and mounted on SuperFrost Plus adhesion slides (J1800BMNZ, Thermo Scientific, Thermofisher Scientific AG, Reinach, Switzerland) and kept at − 20 °C till staining procedure. Paraffin blocks were cut sagitally in 5 µm thick slices using a microtome (RM 2235, Leica biosystems Nussloch GmbH, Nussloch, Germany). For most amino acid transporters staining was performed on cryosections with antigen retrieval using sodium citrate buffer (pH 6.0) for 20 min at 98 °C in the rapid microwave histoprocessor (HistoPRO SW 2.0.0, Milestone medical, Kalamazoo, USA). The sections were incubated for 1 h at room temperature in blocking buffer containing 5% donkey serum (D9663, Sigma-Aldrich Chemie GmbH by Merck, Buchs, Switzerland) and 0.3% Triton X-100. Blocked specimens were then incubated for 1 h at room temperature in incubation buffer (PBS, 1% BSA, 0.3% Triton X-100) containing primary antibodies diluted as indicated in Additional file 2: Table S1. Secondary antibody incubation was performed with donkey anti-mouse DyLight 488 (96875, Abcam, Cambridge Science Park, Milton, Cambridge, UK) and anti-rabbit DyLight 594 (96921, Abcam, Cambridge Science Park, Milton, Cambridge, UK) for 1 h at RT. PBS was used for washes between incubation with primary and secondary antibodies. Nuclear counterstaining was performed by incubation with 2 μg/mL of diamidine-2-phenylindole-dihydrochloride (DAPI) for 10 min at room temperature. Brain sections were mounted with DAKO-Glycergel (C0563, DAKO North America, Carpinteria, USA) and examined under a confocal laser scanning upright microscope Leica TCS SP8 (Leica Microsystems CMS GmbH, Mannheim, Germany) using a 63× objective (oil, numerical aperture of 1.4, pinhole set to 1.0 airy unit). Images were processed and merged by Imaris software (version 7.5.1; bitplane). For LAT2 transporter staining in specimens obtained from LAT2 KO and corresponding age-matched wild-type animals paraffin sections were subjected to deparaffinization (Pathisto AS-2, Pathisto GmbH, Garbsen, Germany), followed by extensive wash in PBS. Antigen retrieval in this case was performed by incubation in 0.1% SDS/PBS for 5 min and subsequent wash in running tap water and PBS. Then sections were blocked for 1 h at room temperature in PBS solution with 5% donkey serum (D9663, Sigma-Aldrich Chemie GmbH by Merck, Buchs, Switzerland) and subsequently incubated overnight in solution containing anti-LAT2 antibodies (1:1000), 1% BSA and 0.02% Triton-X 100. Specimens were washed twice in hyper-PBS (doubled concentration of NaCl, 274 mM) and once in PBS followed by incubation in solution containing secondary anti-rabbit DyLight 488 antibodies diluted 1:500 and DAPI. Afterwards samples were mounted with DAKO-Glycergel and staining was analyzed on a Leica TCS SP8 confocal laser scanning microscope (Leica) using a 63× objective lens (pinhole 1.0, numerical aperture 1.4). Typically stacks of 4 to 8 images (512 × 512) were taken and analyzed at 122 nm intervals through z axis of a section. Alternatively a Nikon Eclipse TE300 epifluorescence microscope (Nikon Instruments Inc, Melville, NY) equipped with a DS-5M Standard charge-coupled device camera (Nikon Instruments Inc) was used. Confocal images were processed using the software Imaris (Bitplane, Zurich, Switzerland). Images with LAT2 staining in CPs of wild-type animals vs LAT2 KO were merged using overlay function in Photoshop 9.

### Choroid plexus isolation

Animals were anesthetized with a ketamine (100 mg/kg) and xylazine (10 mg/kg) cocktail administrated IP, and choroid plexuses were rapidly removed from four ventricles of each animal under stereomicroscope Olympus (SZX10, Volketswil, Switzerland) as described by Bowyer [26]. The rest of each brain (cerebrum and cerebellum separately; referred as total brain) was cut into small pieces (~ 30 mg) and these samples were used later as purity control of isolated CPs. All samples were snap frozen in liquid nitrogen and stored at − 80 °C till further analysis.

### RNA isolation and cDNA synthesis

Total RNA from individual CPs and total brain was isolated with Trizol (15596026, Thermofisher Scientific AG, Reinach, Switzerland) according to the manufacturer protocol followed by purification on RNeasy Micro (74004) or Mini columns (74106, Qiagen AG, Hombrechtikon, Switzerland). Total RNA was quantified using NanoDrop ND 1000 spectrophotometer (Thermo Fisher Scientific Wilmington, USA) and quality was determined using the Agilent 2100 Bioanalyzer (Agilent Technologies, Santa Clara, CA, USA). Only samples with RIN values ≧ 8.0 were used for reverse transcription. The cDNA was synthesized from 100 ng (5 ng/μL) of total RNA using qScript cDNA Synthesis Kit (95047-100, Quantabio, Beverly, USA) according to the manufacturer’s protocol. Quantitative real-time PCR reactions (qRT-PCR) with 10 ng of cDNA as template were performed using the Taq-Man Universal PCR master mix (4304447, Thermofisher Scientific AG, Reinach, Switzerland) in triplicates. In each reaction mixture eukaryotic 18S rRNA endogenous control (4310893E, Thermofisher Scientific AG, Reinach, Switzerland) was included, while cDNA produced without RT enzyme were used as the negative control for each gene. All reactions were carried out in MicroAmp Fast Optical 96-Well Reaction Plates (4346906 Thermofisher Scientific AG, Reinach, Switzerland) using the Fast Real Time PCR System 7500 (Applied Biosystems) with the following parameters: an initial step at 50 °C for 2 min, denaturation at 95 °C for 10 min for polymerase activation followed by 45 cycles with denaturation step at 95 °C for 15 s and annealing/extension at 60 °C for 1 min. Primers and probes were either previously described or designed at Universal probe Library Assay Design Center Roche [27] and listed in Additional file 2: Table S2. Prior to usage, the specificity of all newly designed primers was tested on cDNA samples obtained from several different organs and in each case a single product of expected size was observed. Probes were labeled with reporter dye VIC or FAM at 5′ end and quencher dye TAMRA no dye at 3′ end. Relative expression of each gene of interest was calculated based on the comparative ΔC_T_ method according to the formula: relative expression = 2^−ΔCT^, where ΔCT = average C_T_ value of gene of interest − average C_T_ value of housekeeping gene, where 18S rRNA was used as housekeeping gene. C_T_ values of 18S rRNA were between 7.2 and 12.5. The ones of amino acid transporter mRNAs with a relative expression > 2 × 10^6^ ranged from 24.1 (*Slc38a3*) to 30.3 (*Slc1a3*).

### Amino acid measurements

Measurements of AA concentrations were performed at the Functional Genomic Centre Zurich.

Amino acid concentrations were determined in samples using Mass Track Amino Acid Analysis Application Solution (Waters, Milford, USA) by ACQUITY UPLC according to the manufacturer’s protocol. CSF samples were analyzed directly and for plasma samples deproteinization 1:1 with 10% SSA (sulfosalicylic acid) was performed prior to AA measurements. Plasma samples after precipitation with 10% SSA were diluted 10 times with borate buffer (500 mM, pH 9), precipitated with methanol (5 times) and then analyzed.

### Statistical analysis

Statistical analysis was performed using GraphPad Prism 5.0 (GraphPad Software, USA). Un-paired t-test and one-way analysis of variance (ANOVA) Dunnett (or Bonferroni) post-test were performed for qRT-PCR data and amino acids measurements. All data are presented as mean ± SD or mean ± SEM. Statistical significance was accepted at a level of significance *p* < 0.05 or as indicated.

## Results

### Amino acid transporters expressed in choroid plexus

**Fig. 1:**
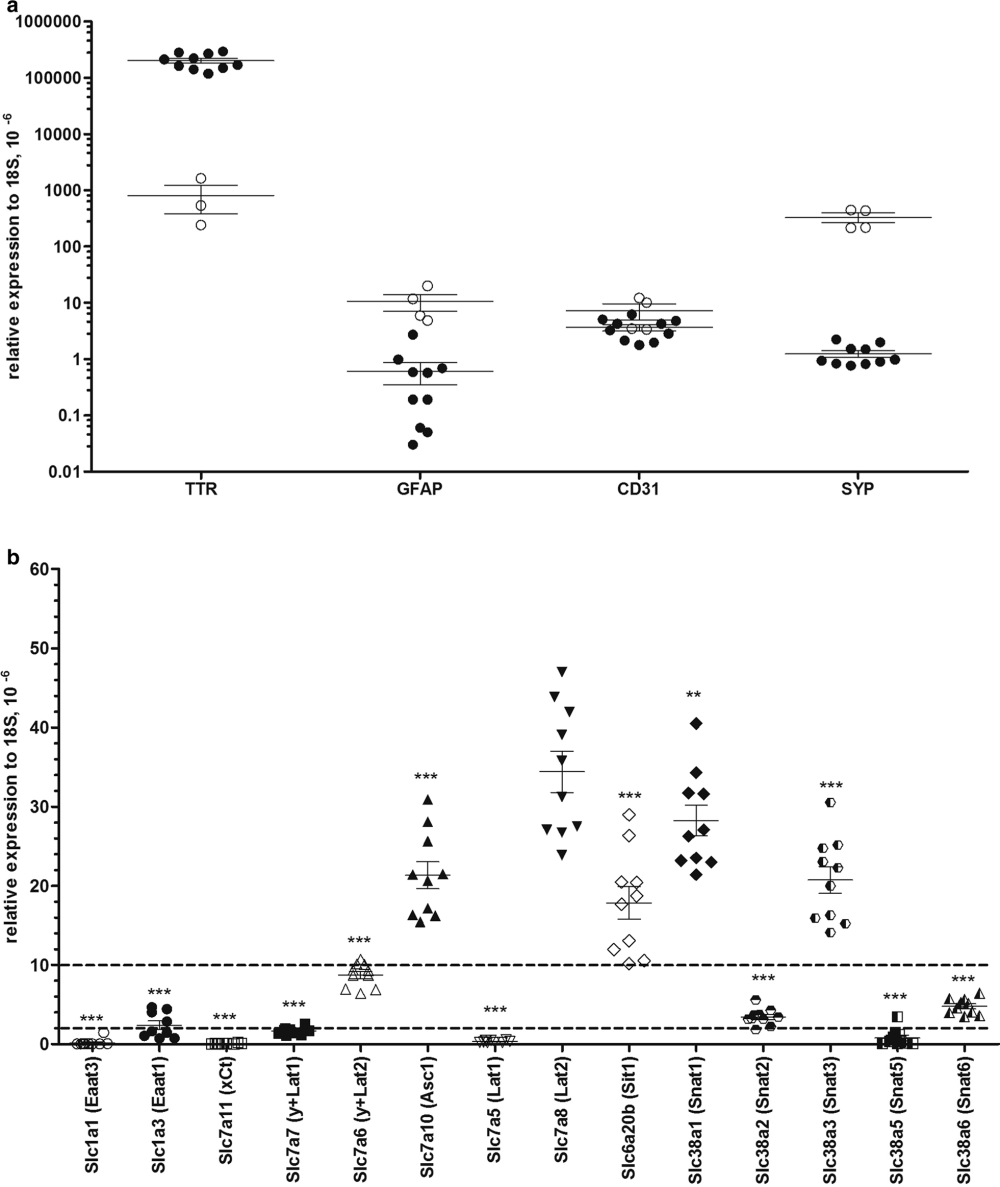
Amino acid transporters expressed in isolated choroid plexuses. **a***Ttr*, *Gfap*, *Cd31* and *Syp* mRNAs in isolated choroid plexuses (closed bars) and cerebrums (open bars) in 3 independent experiments. Data are indicated (mean ± SEM, n = 10). Statistical analysis was performed by unpaired t-test and statistically significant changes are indicated as ***p < 0.001, **p < 0.01. **b** Presence of 14 selected *Slc* genes, which products involved in AA transport, was tested on mRNA level in 3 independent experiments; data presented as mean ± SEM, n = 10. Statistical analysis was performed by ANOVA followed by Dunnet’s post-test and statistically significant differences relative to *Lat2* (*Slc7a8*) mRNA are indicated as ***p < 0.001, **p < 0.01

To study the expression of specific amino acid transporters in CP, we tested first their mRNA levels, although they are known not to correlate with protein expression. However, the presence of an mRNA is per se a prerequisite for the expression of its protein product. We tested initially the purity of the CPs isolated from the four ventricles of each individual animal by measuring the mRNA level of four cell specific markers by qPCR, transthyretin (*Ttr*) as choroidal marker, glial fibrillary acidic protein (*Gfap*) for astrocytes, platelet endothelial cell adhesion molecule-1 (*Pecam 1 or Cd31*) for brain endothelial cells and synaptophysin (*Syp*) for neurons (Fig. 1a). The level of *Gfap* and *Syp* mRNAs were strongly decreased in choroid plexuses when compared to cerebral samples isolated from the same animals (by ~ 91% and ~ 99%. respectively), while the level of *Cd31* mRNA was only halved (~ 46%), reflecting the expected presence of vascular endothelial cells in the choroid plexus samples. Since *Ttr* mRNA was increased ~ 330-fold in isolated choroid plexus compared with cerebral samples, we considered that the enrichments was sufficient and proceeded with a first experiment in which a set of 32 selected *Slc* transcripts encoding AATs (out of 66 known amino acid transporters including intracellular ones [28]) were tested in three animals (Additional file 1: Figure S1). Based on expression values calculated relative to the endogenous reference 18S rRNA, the tested gene products were arbitrarily assigned into three groups with different expression levels: 22 with low (0–2 * 10^−6^ relative to 18S), 5 with moderate (2–10 * 10^−6^ relative to 18S) and 5 with high (> 10 * 10^−6^ relative to 18S) expression level. Taking into account data available in the literature [18–20, 29] and the results of our first experiment, we selected 14 amino acid transporter mRNAs (*Slc1a1*, *Slc1a3*, *Slc6a20b*, *Slc7a5*, *Slc7a6*, *Slc7a7*, *Slc7a8*, *Slc7a10*, *Slc7a11*, *Slc38a1*, *Slc38a2*, *Slc38a3*, *Slc38a5* and *Slc38a6*) for a detailed investigation that involved ten different animals measured in three independent experiments (Fig. 1b). In agreement with previous studies we confirmed a significant mRNA expression for *Slc6a20b* (SIT1), *Slc7a10* (ASC1) and *Slc38a3* (SNAT3) [18–20]. Additionally we found highest mRNA expression levels for two other amino acid transporters, namely *Slc7a8* (LAT2) and *Slc38a1* (SNAT1), actually in contrast to a previous study that had reported lower than average levels [20]. The mRNAs of the *Slc38* family members *Slc38a2* (SNAT2) and *Slc38a6* (SNAT6), the y^+^L system member *Slc7a6* (y^+^LAT2) and the Glu transporter *Slc1a3* (EAAT1 or GLAST) were found moderately expressed, while the mRNAs of *Slc1a1* (EAAT3), *Slc7a5* [large neutral amino acid 1 (LAT1)], *Slc7a7* (y^+^LAT1), *Slc7a11* (xCt) and *Slc38a5* (SNAT5) were expressed at a low level.

### Subcellular localization of amino acid transporters in choroid plexus epithelium

Next, we aimed to localize amino acid transporters highly expressed at the mRNA level, on choroid epithelial cell membranes using immunofluorescence.

**Fig. 2:**
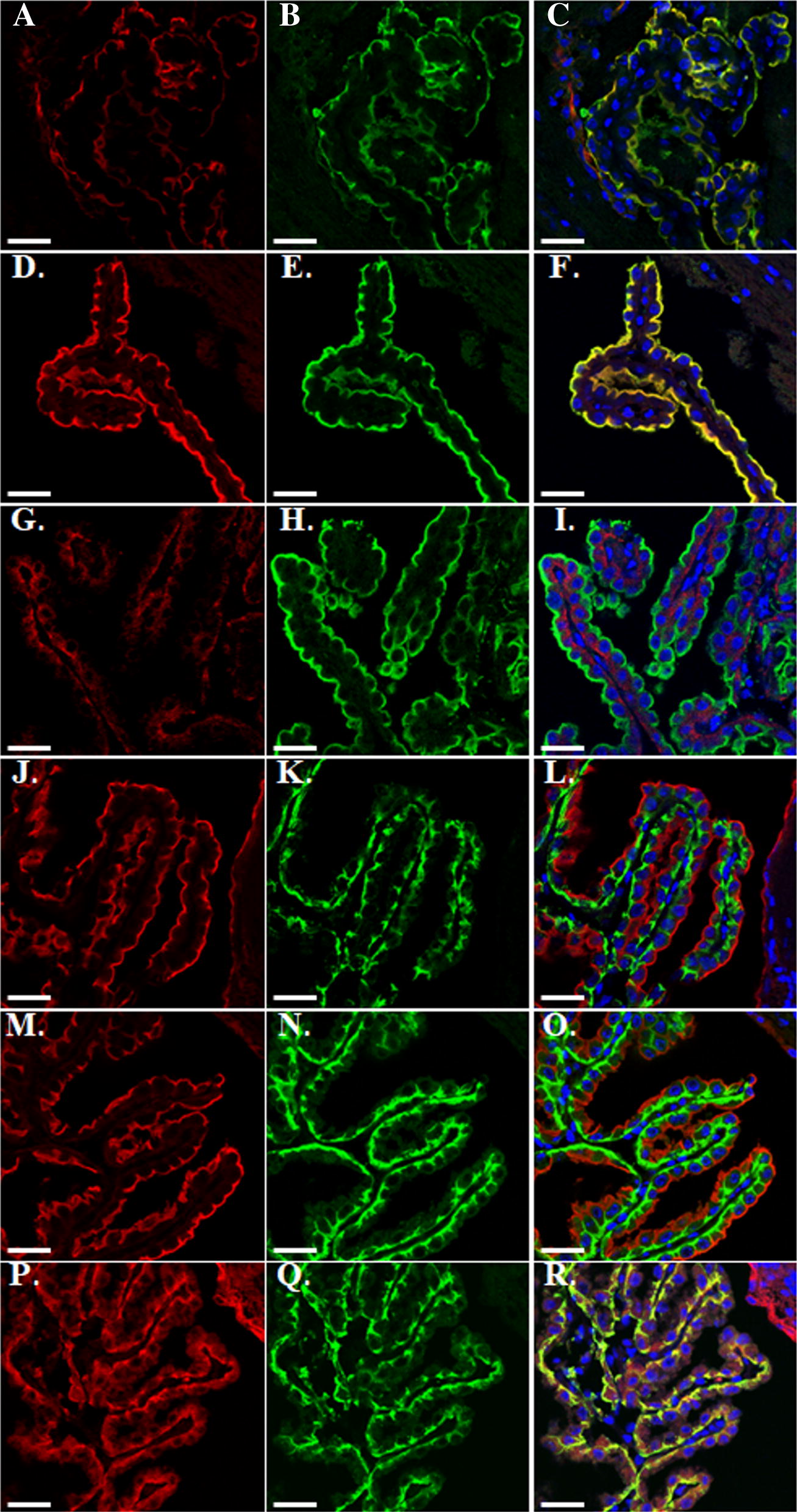
Distribution of three selected AATs in mouse choroid plexuses. Mouse brain cryosections were co-stained with an apical (CSF-facing) membrane marker Na^+^-K^+^-ATPase α (**b**, **e**, **h**) or a basolateral (blood-facing) membrane marker AE2 (**k**, **n**, **q**) and amino acid transporters SNAT3 (**a**, **j**), LAT2 (**d**, **m**), SNAT1 (**g**, **p**); markers are shown in green, AATs in red, nuclei were visualized by DAPI staining in blue. Scale bar is 30 µm

We chose to use only custom-made antibodies the specificity of which had been previously validated in transfected cells or mouse tissue (brain, kidney and cochlea), specifically anti-SNAT3 (Fig. 2a, j), anti-LAT2 (Fig. 2d, m) and anti-SNAT1 (Fig. 2g, p) antibodies [30–34]. The fact that we localized only these three amino acid transporters may be considered as a limitation in view of the larger number of transporters detected at the mRNA level, but since protein localization studies are per se prone to artefacts (cross-reactivity etc.), including only these three increased the reliability of our results.

As a marker for the luminal, CSF-facing membrane of the choroid plexus epithelial cells we used an antibody recognizing the Na^+^, K^+^-ATPase α subunit (isoforms α1–3) and for the basolateral, blood-facing side an antibody recognizing the anion exchanger 2 (AE2). These localizations correspond to the so-called inverse polarization of the choroid plexus epithelium [2, 35, 36]. Staining of adult mouse brain sections revealed in CP clear SNAT3 colocalization with the Na^+^, K^+^-ATPase α subunit (Fig. 2a–c), but not with AE2 (Fig. 2j–l) and interestingly, the same localization pattern was demonstrated for LAT2 (Fig. 2d–f, m–o). While SNAT1 transporter was visualized solely on the basolateral membrane co-localizing with AE2 and resulting in an evident yellow staining (Fig. 2g–i and m, q, r). Unfortunately, we have not been able to observe any reliable signal for the two other amino acid transporters highly expressed in choroid plexus at the mRNA level, ASC1 and SIT1, using commercially available or in-house produced antibodies.

### Alterations in CSF amino acids content of LAT2 knockout animals

**Fig. 3:**
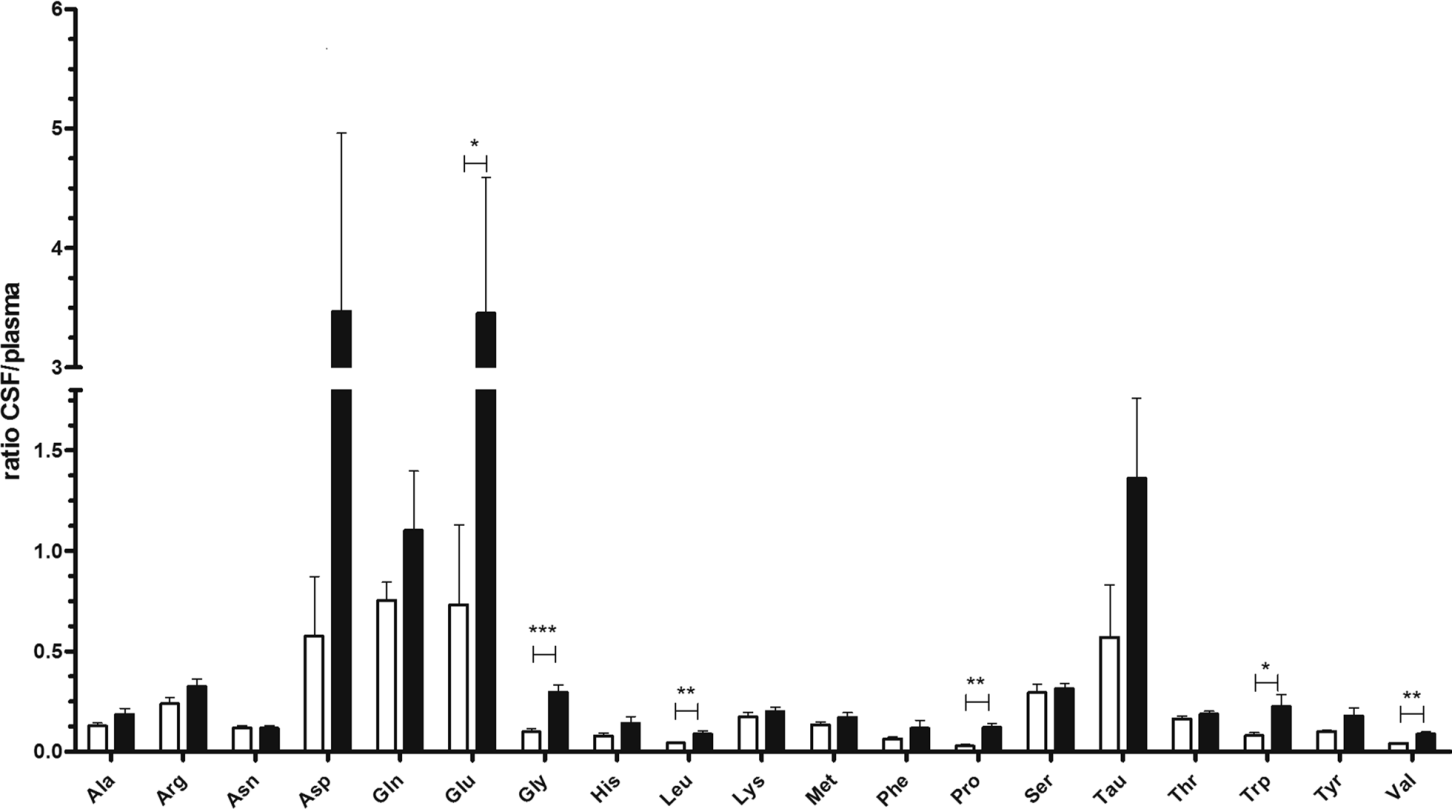
CSP/plasma ratios of amino acids. Amino acids were measured in CSF and plasma samples obtained from LAT2 KO (n = 4; closed bars) and age-matched wt (n = 4; open bars) animals. Data are shown as mean ratios ± SEM and compared with unpaired two-tailed t-test; statistically significant changes are indicated as ***p < 0.001, **p < 0.01, *p < 0.05

Considering the high level of LAT2 expression in CP, we examined the impact of LAT2 ablation on AA concentrations in CSF of *Lat2* KO animals [33]. We confirmed ablation of LAT2 transporter in CP on mRNA and protein levels (Additional file 1: Figure S2A, B) and measured amino acid levels in plasma and CSF samples. Previously Braun et al. had reported elevated levels of 8 amino acids (Ala, Ser, Gly, Thr, Glu, Asp and Lys) for the serum of LAT2 knockout (KO) animals [37], however these alterations were not reproduced in our experiments using another LAT2 knock-out model (Additional file 2: Table S3) [32, 33]. Therefore, we compared the CSF/plasma ratio of each out of 19 detected amino acids [18 proteinogenic AA (all except Cys and Ile) and Tau] between wild type and LAT2 KO animals. Raised CSF/plasma ratios were detected for at least six amino acids (other possible increases were not significant): the large neutral branched chain and aromatic amino acids Leu (p < 0.01), Val (p < 0.01) and Trp (p < 0.05), the inhibitory neurotransmitter Gly (p < 0.001), the imino acid proline (Pro) (p < 0.01), and the excitatory amino acid Glu (p < 0.05) (Fig. 3). Interestingly, the latter three amino acids are not influx substrates of LAT2 [38], suggesting a possible functional cooperation of LAT2 with other amino acid transporters.

## Discussion

In this study, we performed a careful comparative analysis of AATs expressed in mouse choroid plexus. Before discussing the functional implications of our new findings below, we first compare here our new data with previously published ones. Importantly, in addition to the previously reported high mRNA levels of SNAT3 (system N Gln-Na^+^ symporter/H^+^ antiporter) (*Slc38a3*), ASC1 (Ala-Ser-Cys (cysteine) antiporter 1) (*Slc7a10*) and SIT1 (imino acid-Na^+^ symporter) (*Slc6a20b*) [18–21], we detected in choroid plexus also high mRNA levels of the neutral amino acid antiporter LAT2 (*Slc7a8*) and of the system A Gln-Na^+^ symporter SNAT1 (*Slc38a1*). In contrast, in a previous mouse CP transcriptome analysis performed by Marques and colleagues, LAT2 (*Slc7a8*) mRNA had been considered to be expressed at a level lower than average, similar to TAUT (*Slc6a6*), GLYT1 (*Slc6a9*) and LAT1 (*Slc7a5*) [20]. Our well controlled qPCR analysis revealed however that besides the highly expressed mRNA of LAT2 (*Slc7a8*), the three other mRNAs (*Slc6a6*, *Slc6a9*, *Slc7a5*) were hardly detectable. These differences between the amino acid transporter mRNA expression levels measured in the present study and the previously published results are suggested to be due to different methodological approaches, in particular regarding the quantitation method. In support of our findings, another more recent study also suggested a significant expression of LAT2 mRNA in choroid plexus epithelial cells, but without localization by immunohistochemistry [33]. Protein expression of LAT2 had been detected previously by proteome analysis of whole CP but not in BAB [16]. The high expression level of this neutral amino acid antiporter was confirmed by the present study and its localization to the luminal membrane of CP epithelial cells determined. We also detected a moderate expression of y^+^L system member y^+^LAT2 (*Slc7a6*) mRNA in murine CP that corresponds to previously reported in situ hybridization results [19]. In agreement with an earlier study by Lee et al. [39], also Glu transporter EAAT1/GLAST (*Slc1a3*) mRNA was detected in our study. Recently another glutamate transporter, EAAT3 (*Slc1a1*), was suggested to be the main Glu transporter in CP based on immunofluorescence and transport studies made in rat [23]. However, we failed to detect a substantial expression of this transporter on the mRNA level in the current investigation. It is not clear whether these seemingly opposing results are due to a difference between rats and mice or to some technical reasons. The function of the choroid plexus epithelium is key to understand the role of amino acid transporters expressed in this highly specialized epithelium.

Next to the support of cell housekeeping functions, choroid plexus amino acid transporters are required for the transepithelial transport that controls the amino acid concentration levels in CSF. This task is quantitatively important, as the choroid plexus secretes fluid at a rate, which is higher than that of any other secretory epithelia [2]. Additionally, the amino acid concentration in CSF is maintained at a stable level that is for all amino acids 2–20-fold lower than in plasma, but for Gln, the concentration of which is only slightly lower in CSF compared to plasma [25]. The structural organization of this epithelium is well adapted to its major secretory task and is characterized, unlike classical epithelia of intestine and kidney, by an “inverse” polarity of Na^+^, K^+^-ATPase, NKCC1, KCC4 and NHE1 expression which localize to the luminal, CSF facing membrane [2]. The directed transepithelial ion transport, in particular of Na^+^, Cl^−^ and HCO_3_^−^, is critical for the proper water transport and thus, CSF production, but not fully understood. Clear is that the driving force for this transport is generated by the luminal Na^+^, K^+^-ATPase, which actively pumps Na^+^ ions from the choroid plexus cells into the CSF. It has also been shown that several antiporters and symporters take advantage of the electrochemical driving force provided by the Na^+^ gradient to co-transport and/or exchange Cl^−^, K^+^, HCO_3_^−^, H^+^ etc. and thereby play important roles [3]. In our amino acid transporter localization study using immunofluorescence imaging, we showed that the Na^+^ symporter SNAT1 (*Slc38a1*) that co-transports neutral non-essential amino acids, in particular Gln with Na^+^, localizes to the basolateral membrane (blood side) of choroid plexus cells. We also showed that in contrast SNAT3 (*Slc38a3*), another Na^+^ dependent symporter that additionally exchanges H^+^, localizes to the luminal CSF-facing membrane of choroid plexus epithelial cells together with the neutral amino acid exchanger LAT2 (*Slc7a8*).

**Fig. 4:**
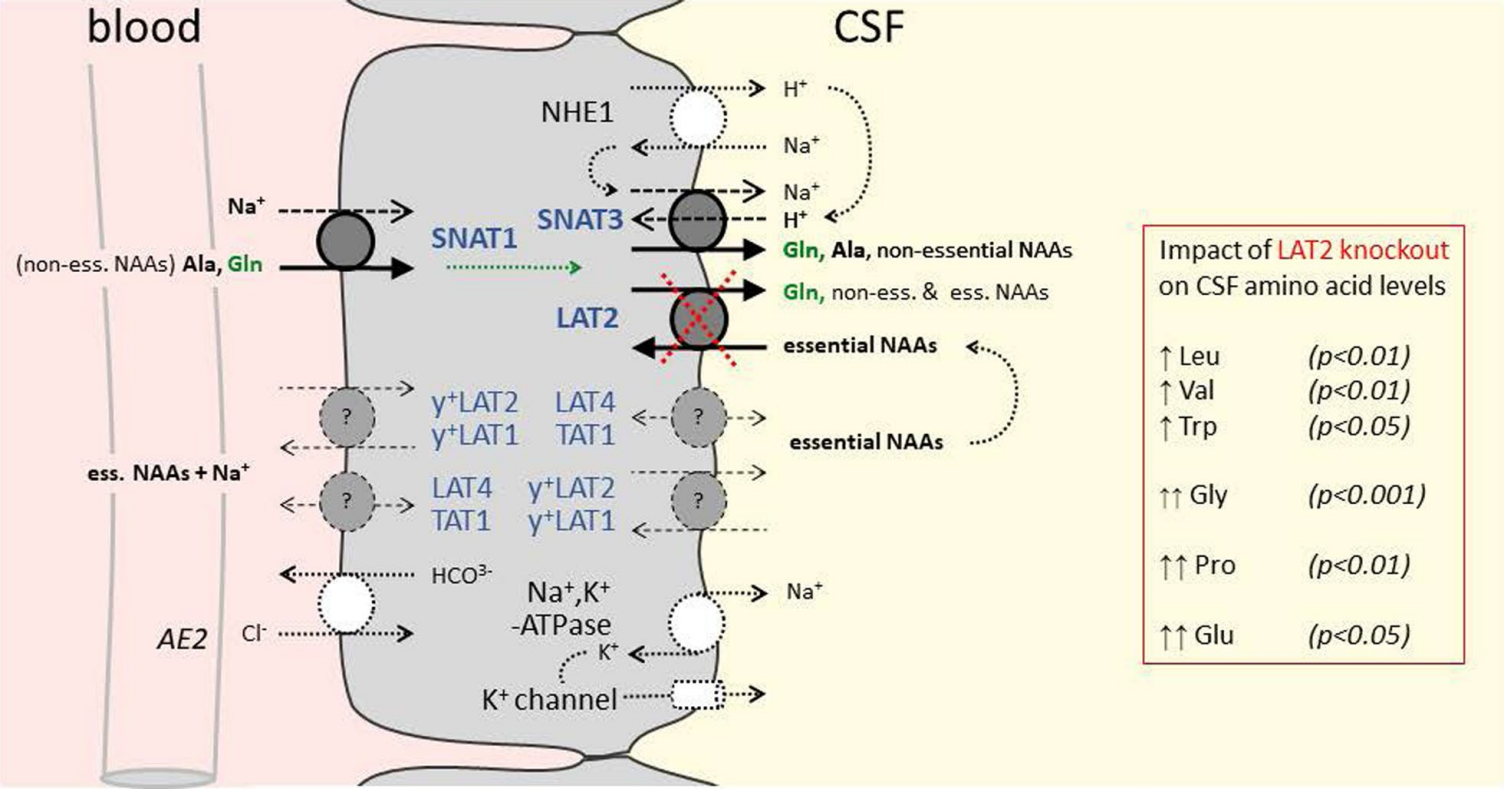
Schematic representation of CP epithelial cell showing amino acid transporters localized in this study. The amino acid—Na^+^ symporter SNAT1 (*Slc38a1*) is shown together with the marker protein anion exchanger 2 (AE2) at the vascular basolateral side of the CP epithelium. The amino acid antiporter LAT2 (*Slc7a8*) and the neutral amino acid—Na^+^ symporter/H^+^ antiporter SNAT3 (*Slc38a3*) are shown together with the marker protein Na^+^, K^+^-ATPase and a K^+^ channel at the CSF-facing luminal side of the CP epithelium. The sodium/proton exchanger NHE1 which might cooperate with SNAT3 to allow the efflux of Gln into CSF is also represented. Additionally, shown in dotted lines are amino acid transporters of which in this study only the mRNA has been detected in CP [moderately expressed antiporter y^+^LAT2 (*Slc7a6*) and low level expressed antiporter y^+^LAT1 (*Slc7a7*) and uniporters LAT4 (*SLC43a2*) and TAT1 (*Slc16a10*)] which may participate to the passage of neutral essential amino acids into CSF. Other amino acid transporters clearly detected at the mRNA level in the present study but not yet localized to a specific choroid plexus epithelium membrane [SIT1 (Slc6a20b) and ASC1 (*Slc7a10*) (high mRNA levels) as well as SNAT2 (*Slc38a2*), SNAT6 (*Slc38a6*) and EAAT1 (*Slc1a3*) (moderate mRNA levels)] and other potentially important amino acid transporters not tested in the present study are not included in the schema. CSF amino acids the concentration ratios of which (CSF/plasma, see Fig. 3) are changed ≥ 2-fold in LAT2 (*Slc7a8*) knockout mice are indicated with Students *t*-test *p*-values; (non-)ess. *NAAs* (non-)essential neutral amino acids

It appears thus that the neutral amino acid-Na^+^ symporter SNAT1 drives the basolateral uptake of non-essential neutral amino acids, in particular of Gln, into choroid plexus epithelial cells (see schema presented in Fig. 4). With its relatively low apparent K_m_ of ~ 300 µM [40] for Gln (vs Gln plasma levels of ~ 700 µM) and the vectorial flux of Na^+^ from blood into CSF, this basolateral Na^+^ symporter is indeed ideally suited for the uptake of a controlled amount of Gln. This amino acid is nearly as concentrated in CSF as in blood and, thus needs to be efficiently transported across the choroid plexus epithelium. As regards the luminal release of Gln into the CSF, we propose that SNAT3 functions as a main luminal efflux pathway, by co-transporting it with Na^+^ in exchange for an H^+^, the recycling of which might be via the parallel localized sodium/proton exchanger NHE1. The transport direction of Gln via SNAT3 indeed strongly depends on the local chemical Na^+^, H^+^ and Gln driving forces. For instance, electroneutral efflux of Gln from astrocytes has been shown to take place via this transporter during the Glu–Gln cycle [41, 42]. However, the amino acid levels measured in the CSF of LAT2 knockout mice suggest that also LAT2 participates to the luminal efflux of Gln. Indeed, in the absence of LAT2 the concentration of essential amino acids was strongly increased in CSF, whereas the non-essential neutral amino acids transported by SNAT3, like for instance Gln, were nearly normal. This suggests the possibility that normally the efflux of some Gln and other neutral non-essential amino acids into the CSF via the antiporter LAT2 drives in exchange the uptake of essential amino acids back from the CSF into choroid plexus cells. The increased level of essential amino acids observed in the CSF of LAT2 KO mice indicates that they must be transported presumably also across CP cells independent of LAT2. Thus, we suggest that other amino acid transporters detected at the mRNA level in our study, but not yet localized in CP cells at the protein level, for instance the antiporter y^+^LAT2 and/or y^+^LAT1 and the uniporters LAT4 and TAT1, may be involved (Fig. 4). The observation that the excitatory amino acids Glu and Asp and the imino acid Pro that are not LAT2 substrates and also the poor LAT2 uptake substrate Gly were most highly increased in CSF of LAT2 KO mice, is not explained by our schema presented in Fig. 4 and suggests a functional cooperation of this exchanger with other amino acid transporters, in addition to SNAT3. A caveat concerning the present discussion about the effect of LAT2 deletion is the fact that we did not test in these mice whether the expression of other CP amino acid transporters was affected by the lack of LAT2 and additionally influenced CP amino acid transport and CSF amino acid levels.

## Conclusions

The results of this study suggest that the Na^+^-symporter SNAT1 (*Slc38a1*) plays a central role for the active transport of non-essential neutral amino acids, in particular of Gln, from the blood into CP epithelial cells and that SNAT3 (*Slc38a3*) and LAT2 (*Slc7a8*) are key for their luminal release into CSF. With its antiporter function, LAT2 appears thereby to reuptake essential neutral amino acids from the CSF and thus to participate to the maintenance of the amino acid concentration gradient between plasma and CSF [4, 5]. Next to these three amino acid transporters of which we have determined the polarity of localization in CP epithelial cells, other amino acid transporters need to cooperate for the transfer of the full set of amino acids across the blood–CSF barrier. Based on their mRNA expression level, their known transport function and our published experience with kidney proximal tubule epithelial amino acid transport, we postulate, that the antiporter y^+^LAT2 (*Slc7a6*) and the lower expressed (at mRNA level) antiporter y^+^LAT1 (*Slc7a7*) and the uniporters LAT4 (*SLC43a2*) and TAT1 (*Slc16a10*) play important roles (Fig. 4) [32]. A limitation of our study and of our speculative transport schema shown in Fig. 4 is that amino acid transporters, the mRNA of which we did not test, may play important functional roles. This could for instance be the case for transporters encoded by *Slc6a14*,*15*,*17*,*18*,*19*, *Slc7a9*,*13*, *Slc36a1*-*4* and *Slc43a3* that displayed, in an earlier CP microarray study, expression levels > 6.0, potentially compatible with a functionally relevant transporter expression [20].

Taken together, the results of this study represent to our knowledge a first description of how neutral amino acids, and in particular glutamine, are potentially transported across choroid plexus epithelial cells into the CSF. This amino acid transport across the CP, together with that across the blood brain barrier, is crucial for brain amino acid homeostasis and thus brain function.

## Supplementary information

**Additional file 1: Figure S1.** Expression profile of 32 *Slc* genes in isolated choroid plexuses (n = 3). **Figure S2.** Assessment of LAT2 knockout in the brain tissue.

**Additional file 2: Table S1.** Antibody dilutions used. **Table S2.** Primers and probes used for qRT-PCR. **Table S3.** AA concentrations measured in CSF and plasma samples of wt and Lat2−/− animals.

## Abbreviations

AAs: Amino acids
AATs: Amino acid transporters
ALS: Amyotrophic lateral sclerosis
AD: Alzheimer disease
Ala: Alanine
Asp: Aspartate
BAB: Blood–arachnoid barrier
BBB: Blood–brain barrier
BCSF: Blood–CSF barrier
CSF: Cerebrospinal fluid
CP: Choroid plexus
GFAP: Glial fibrillary acidic protein
Gln: Glutamine
Glu: Glutamate
Gly: Glycine
LAT1: Large neutral amino acid transporter type 1
LAT2: Large neutral amino acid transporter type 2
Leu: Leucine
NAAs: Neutral amino acids
Orn: Ornithine
Pecam 1 (CD31): Platelet endothelial cell adhesion molecule 1
Pro: Proline
Ser: Serine
SNAT1: Sodium-dependent amino acids co-transporter, system A member
SNAT2: Sodium-dependent amino acids co-transporter, system A member
SNAT3: Sodium-dependent amino acids/H + co-transporter
SYP: Synaptophysin
Trp: Tryptophan
Thr: Threonine
TTR: Transthyretin
Val: Valine
