# Supplementary material for: Choroid plexus LAT2 and SNAT3 as partners in CSF amino acid homeostasis maintenance
Source: Fluids Barriers CNS. 2020 Feb 11;17:17. doi: 10.1186/s12987-020-0178-x (PMC7014681; doi:10.1186/s12987-020-0178-x)
Supplement: Supplementary file 1 — Additional file 1: Figure S1. Expression profile of 32 Slc genes in isolated choroid plexuses (n = 3). Figure S2. Assessment of LAT2 knockout in the brain tissue. [file 12987_2020_178_MOESM1_ESM.pdf]

Choroid plexus LAT2 and SNAT3 as partners in CSF amino acid homeostasis maintenance

Elena Dolgodilina, Simone M. Camargo, Eva Roth, Brigitte Herzog, Virginia Nunes, Manuel Palacín, Francois Verrey

Additional File 1
Supplementary Figures

Figure Lengends

Figure S1. Expression profile of 32 *Slc* genes in isolated choroid plexuses (n=3).

For each AA transporter gene, mRNA levels were measured by qPCR relative to 18S rRNA and presented as mean ± SD.

Figure S2 Assessment of LAT2 knockout in the brain tissue

A. Significant reduction of *Lat2* mRNA in CP (white bars) and cerebrum (dotted grey bars) of LAT2 KO mice compared to wild-type samples. Data shown as mean ± SD and compared with unpaired t-test, n=5 for KO's and wt CP, 4 for wt cerebrum, ***p<0.001.
B. Representative staining of LAT2 transporter (green) in wt and LAT2 KO CP paraffin sections. Nuclei are counterstained with DAPI (blue). Scale bar is 50 µm.

Figure S1.

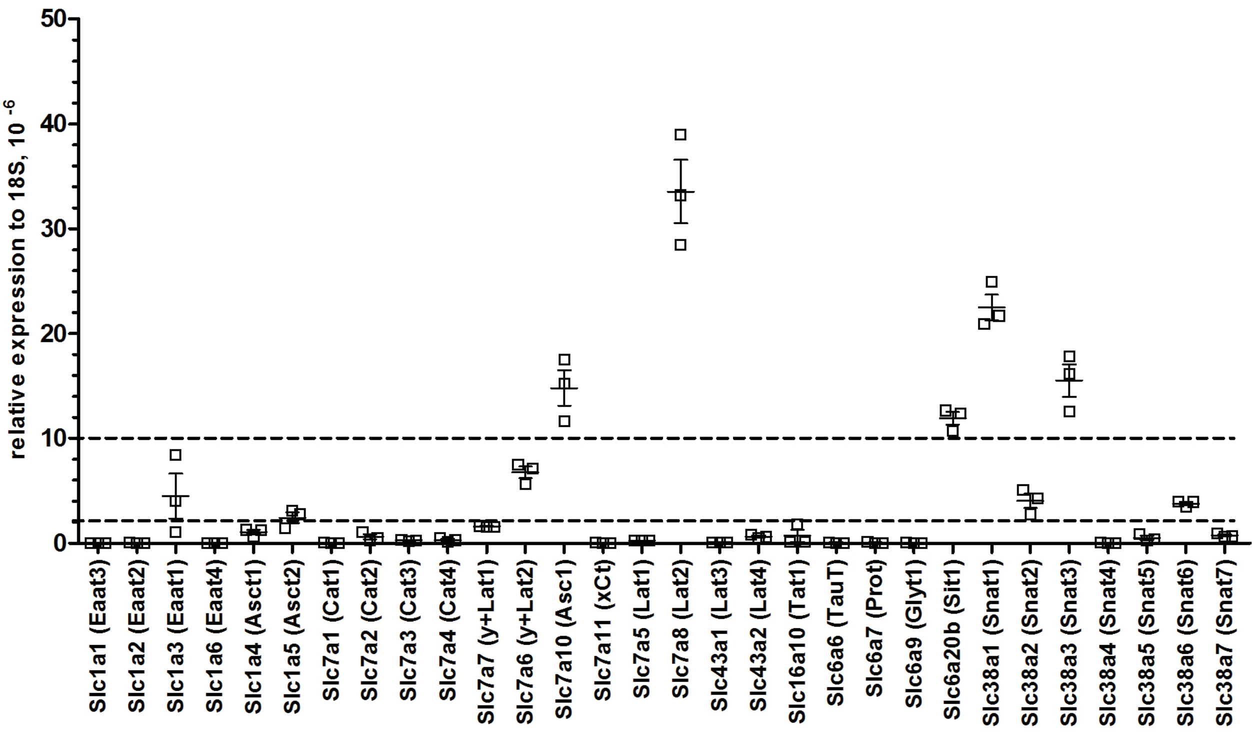

50
40
30
20
10
0
relative expression to 18S, 10 -6
Slc1a1 (Eaat3)
Slc1a2 (Eaat2)
Slc1a3 (Eaat1)
Slc1a6 (Eaat4)
Slc1a4 (Asct1)
Slc1a5 (Asct2)
Slc7a1 (Cat1)
Slc7a2 (Cat2)
Slc7a3 (Cat3)
Slc7a4 (Cat4)
Slc7a7 (y+Lat1)
Slc7a6 (y+Lat2)
Slc7a10 (Asc1)
Slc7a11 (xCt)
Slc7a5 (Lat1)
Slc7a8 (Lat2)
Slc43a1 (Lat3)
Slc43a2 (Lat4)
Slc16a10 (Tat1)
Slc6a6 (TauT)
Slc6a7 (Prot)
Slc6a9 (Glyt1)
Slc6a20b (Sit1)
Slc38a1 (Snat1)
Slc38a2 (Snat2)
Slc38a3 (Snat3)
Slc38a4 (Snat4)
Slc38a5 (Snat5)
Slc38a6 (Snat6)
Slc38a7 (Snat7)

Figure S2.

A.

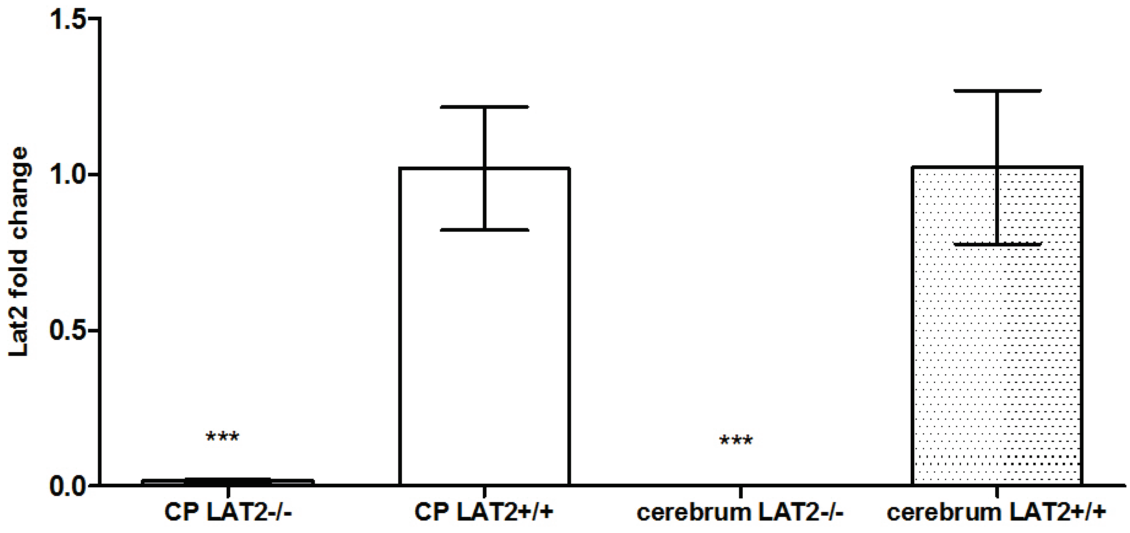
Lat2 fold change
1.5
1.0
0.5
0.0
***
***
CP LAT2-/-
CP LAT2+/+
cerebrum LAT2-/-
cerebrum LAT2+/+

B.

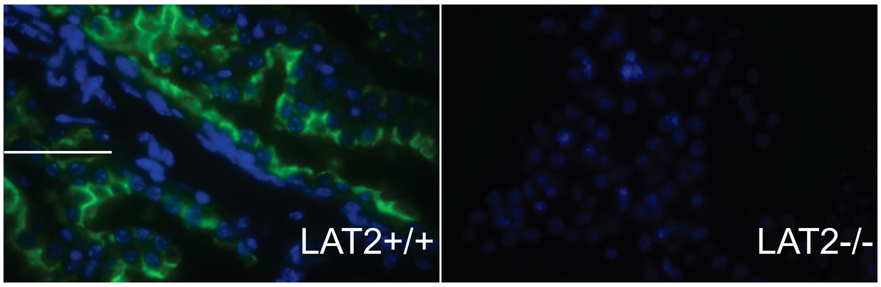
LAT2+/+
LAT2-/-
