# Supplementary material for: Choroid plexus LAT2 and SNAT3 as partners in CSF amino acid homeostasis maintenance
Source: Fluids Barriers CNS. 2020 Feb 11;17:17. doi: 10.1186/s12987-020-0178-x (PMC7014681; doi:10.1186/s12987-020-0178-x)
Supplement: Supplementary file 2 — Additional file 2: Table S1. Antibody dilutions used. Table S2. Primers and probes used for qRT-PCR. Table S3. AA concentrations measured in CSF and plasma samples of wt and Lat2−/− animals. [file 12987_2020_178_MOESM2_ESM.pdf]

Choroid plexus LAT2 and SNAT3 as partners in CSF amino acid homeostasis maintenance

Elena Dolgodilina, Simone M. Camargo, Eva Roth, Brigitte Herzog, Virginia Nunes, Manuel Palacín, Francois Verrey

Additional file 2
Supplementary tables

Table S1: Antibody dilutions used

Target protein	Target sequence	Species of target protein	Host	Dilution	Manufacturer
SNAT3 (SLC38A3)	MEIPRQTEMVELVPNGKC (N-terminus)	Mouse	Rabbit	1:100	In house via Pineda
SNAT1 (SLC38A1)	MMHFKSGLELTELQNMTVC (N-terminus)	Mouse	Rabbit	1:100	In house via Abent
LAT2 (SLC7A8)	CPIFKPTPVKDPDSEEQP (C-terminus)	Mouse	Rabbit	1:100, or 1:1000	In house via Eurogentech
AE2 (SLC4A2)	100-255 AA (N-terminus)	Human	Mouse	1:100	Santa Cruz Biotechnology SC-376632
Na^+-K^+-ATPase α (H-3)	551-850 AA	Human	Mouse	1:100	Santa Cruz Biotechnology SC-48345

Table S2: Primers and probes used for qRT-PCR

	Gene	Full name	Accession number		Sequence
**Markers**					
1	*Cd31/Pecam1*	platelet/endothelial cell adhesion molecule 1	NM_008816	forward	TCCCCGAAGCAGCACTCT
				reverse	CCGCAATGAGCCCTTTCTT
				probe	TCAGAGTCTTCCTTGCCCCA
2	*Gfap*	glial fibrillary acidic protein	NM_010277	forward	CACAGCGGCCCTGAGAGA
				reverse	GTCTGCAAACTTAGACCGATACCA
				probe	ACTCAATACGAGGCAGTGGCCACCAGTAA
3	*Syp*	Synaptophysin	NM_009305	forward	GGGCCAATGATGGACTTCCT
				reverse	GGATGAGCTAACTAGCCACATGAA
				probe	CACAGCAGTGTTCGC
4	*Slc2a1*	Glut1	NM_011400	forward	TCGTCGTTGGCATCCTTATTG
				reverse	TGCATTGCCCATGATGGA
				probe	CCAGGTGTTTGGCTTAG
5	*Ttr*	Transthyretin	NM_013697.5	forward	CATGAATTCGCGGATGTG
				reverse	GATGGTGTAGTGGCGATGG
				probe	Roche probe#76

Transporters					
1	*Slc1a1*	Eaat3	NM_009199.2	forward	GGTCTGCGCGCTGTAGTAT
				reverse	TGAGTGACACCAGGCTTGATA
				probe	Roche probe #77
2	*Slc1a2*	Eaat2	NM_001077514	forward	TGCTCATCCTCCCTCTTATCATC
				reverse	GGCCGCTGGCTTTAGCAT
				probe	AGTTTAATCACAGGGTTGTC
3	*Slc1a3*	Eaat1	NM_148938	forward	GAAGCCATCATGCGATTGGT
				reverse	CCCTGCGATCAAGAAGAGGAT
				probe	CGGTGATAATGTGGTATGC
4	*Slc1a4*	Asct1	NM_018861.3	forward	TGGACTCTTTCCTCGATTTACTCA
				reverse	AATCGGTTGCAGACGTAGTGAA
				probe	CCTGTTCCCTTCCAATCTTGTGGTTGC
5	*Slc1a5*	Asct2	D85044	forward	CATCCTGGAAGCAGTCAGCC
				reverse	CACCTTCCACGTTGAGGACA
				probe	TGATCTTGGCCGTGGACTGGCTAGTG
6	*Slc1a6*	Eaat4	NM_009200	forward	CCCCAGGCAGGACTGGTTA
				reverse	TTGTACGAAGTCGGTCAAGGAA
				probe	TGATTGTGCTCACATCCGTCGGC
7	*Slc6a5*	Glyt2	NM_148931.3	forward	ATGTTTGGAGGTTTCCCTACC
				reverse	TCAAGTAAGGGATGAGGAACG
				probe	Roche probe #97
8	*Slc6a6*	Taut	NM_009320	forward	GCAACGTCTGGCGTTTCC
				reverse	AGGCCGCTCCCAAACAG
				probe	TGCGTTCCTCATACCGT
9	*Slc6a7*	Prot	NM_201353.1	forward	GGAAGGGCATCCAGTTCTATC
				reverse	AGCAGCTTCAATCCACACCT
				probe	Roche probe #77
10	*Slc6a9*	Glyt1	NM_008135.4	forward	AATGTCTGGCGTTTCCCATA
				reverse	TGATGAAGTAGGGGAACATGAA
				probe	Roche probe #52
11	*Slc6a20b*	SIT1	NM_011731.3	forward	GGAAGCATGCTTGGAAACACAG
				reverse	AACACCATGCCAACAGCACA
				probe	AGGAGGCCATTTCAGGTTTGGTATGCCTC
12	*Slc7a1*	Cat1	NM_007513.4	forward	GGCAGCTCACGGAGAAAAATT
				reverse	TCCCTCACCGTATTTCACGTT
				probe	TCCTGTAACAACAACGACAC
13	*Slc7a2*	Cat2	NM_007514.3	forward	CATCACTGGCTGGAATCTCATC
				reverse	ACTCCATGCTCTTGCGACACT
				probe	TGTCATATGTCATAGGTACGTC
14	*Slc7a3*	Cat3	NM_007515.2	forward	ACTCCCTGGTGTCCATTTGTG
				reverse	TCTTCAACACTCTTCATTTCCTGATC
				probe	TCTCATCCTCAGATATCA
15	*Slc7a4*	Cat4	NM_144852.3	forward	GGCTCTATCTGTGCCATGAACA
				reverse	GCGCGGCAGGGAGAA
				probe	TCCTGCTCAGCAACC
16	*Slc7a5*	Lat1	NM_011404	forward	GGAGGATGGAACTATCTGAATTTTG
				reverse	GGTTGGTAGAGAGGGTAGTGAAATAGG
				probe	CTACAGGAACCTGCCCCTGGCCA

17	*Slc7a6*	y+Lat2	BC038404	forward<br>reverse<br>probe	TACATCCTGACCAACGTGGC<br>ATGCCGAATGTCTGGTCAGC<br>TCCATAAGAGTGACGCTGTGCCTGTGA
18	*Slc7a7*	y+Lat1	NM 011405	forward<br>reverse<br>probe	AATTCCAGTAGCGGTTGCATT<br>GGAGGTGGCCTTCTCTCGAG<br>TTGCTTTGGTGGGCTCAACGCC
19	*Slc7a8*	Lat2	NM 016972	forward<br>reverse<br>probe	TCCACGTTTGGTGGAGTCAA<br>TCACACAACCGGTACTAGGT<br>CTCCCTCTTCACCTCCTCCCGGCT
20	*Slc7a10*	Asc1	NM_017394.4	forward<br>reverse<br>probe	TGGCTGGAACTTCCTCAACT<br>GATGGCACGAGGTAGGTTCT<br>Roche probe #17
21	*Slc7a11*	xCT	NM_011990.2	forward<br>reverse<br>probe	ACCATCAGTGCGGAGGAG<br>AAGATCGGGACTGCTAATGAGA<br>Roche probe #3
22	*Slc7a12*	Asc2	NM_080852	forward<br>reverse<br>probe	CAGAACCCTGATATACCAAGACCT<br>GGAAAAGTGATAAAGCTATAGATCCAA<br>Roche probe #5
23	*Slc16a10*	TAT1	NM_028247.4	forward<br>reverse<br>probe	CGCCTACGGGGTGCTCTTC<br>ACTCACGATGGGGCAGCAG<br>CGAGCCCACCCACGCTGTCTTG
24	*Slc38a1*	Snat1	AF184240	forward<br>reverse<br>probe	CGCGTGCACACCAAAGTATG<br>AGATTGGCAGGACGGACG<br>TACCAACCATCGCCTTCGCGTTTG
25	*Slc38a2*	Snat2	BC041108	forward<br>reverse<br>probe	CCAATGAGATCCGTGCAAAA<br>TGGACCCAATCCAGCACAAT<br>TCTGTGTTTTCTCCTGAGTGGCATAGTGGTG
26	*Slc38a3*	Snat3	AF159856	forward<br>reverse<br>probe	CGAATCATGCCCACTGACAA<br>AACCGCAGCGAAACAAAGG<br>AGCCTGCAAGATCCACCCCTAAAATCCT
27	*Slc38a4*	Snat4	AK051023	forward<br>reverse<br>probe	AGGGTGCTGAGA AATGCCAA<br>GACCTCAGGGTGGCAGAC AA<br>AATACTTTGTGTTCAATTCCCGGACGGC
28	*Slc38a5*	Snat5	AK054485	forward<br>reverse<br>probe	CCCTCACTGTGCCTGTTGTG<br>CTTGCTTGGAAAGAGCAGCTG<br>CCCTATCCGCCGAGCCCTCCA
29	*Slc38a6*	Snat6	BC043932	forward<br>reverse<br>probe	CGCCGTGCTTCTGACAGCC<br>GGGAATGTCGGATCCAGGA<br>CTTTAATGATGATCCTGTTCTCCAATTACCCGTT
30	*Slc38a7*	Snat7	NM_172758.4	forward<br>reverse<br>probe	GGCTCTGACCCCATACCTTT<br>GGGGAACTCTTGTCAACCTG<br>Roche probe #6
31	*Slc43a1*	Lat3	NM_001081349	forward<br>reverse<br>probe	CCCTGAATGAGAATGCTTCCTT<br>ATGGCATTGGTGAGCTTTTGT<br>AGCACCAAGTTCACTAGACCACGCTACCG
32	*Slc43a2*	Lat4	NM_173388	forward<br>reverse<br>probe	GCTGATTGCATATGGAGCAAGTAAC<br>CGAAGTGAACGTCATGCACAT<br>CTCTCTGTGCTCATCTTTATCGCCTTGGC

Table S3: AA concentrations measured in CSF and plasma samples of wt and Lat2$^{-/-}$ animals

**Amino acids**	**Plasma concentrations, μmol/L ±SD**		**CSF concentrations, μmol/L ±SD**	
	LAT2+/+	LAT2-/-	LAT2+/+	LAT2-/-
Ala	551.84±276.83	685.12±272.34	65.13±17.40	116.60±22.84*
Arg	105.74±22.15	118.54±25.76	24.60±5.35	37.18±6.95*
Asn	45.13±6.20	78.42±13.81**	5.28±0.84	9.09±1.39**
Asp	12.43±5.27	15.25±7.59	5.08±3.39	42.86±28.70*
Gln	738.08±143.83	1103.16±235.80*	553.43±141.16	1191.18±653.97
Glu	24.05±11.86	42.92±37.36	13.29±9.47	97.44±36.85**
Gly	280.23±48.56	344.90±67.12	27.14±9.30	101.53±21.88***
His	76.28±7.56	86.01±17.19	5.76±1.63	11.73±5.77
Leu	134.80±30.83	141.02±26.64	5.52±1.22	12.04±2.28**
Lys	286.73±54.65	318.88±74.51	48.28±13.46	62.87±16.61
Met	60.18±8.20	63.29±10.63	7.97±1.91	10.63±3.46
Phe	70.87±15.26	75.29±19.53	4.30±1.03	8.63±5.79
Pro	83.49±24.56	129.50±31.84	2.57±1.63	14.62±3.26***
Ser	143.03±16.32	181.59±19.50*	41.29±8.70	57.37±14.83
Thr	159.46±12.67	181.22±35.38	26.42±5.75	33.58±9.04
Trp	65.22±9.41	65.21±10.92	5.02±1.36	15.10±10.24
Tyr	73.34±15.35	84.14±27.84	7.35±1.18	13.55±6.31
Val	214.17±39.40	244.16±31.09	8.34±1.32	20.86±2.38***
Ile	88.20±20.92	94.97±16.00	not reliably detected in CSF	
Tau	486.92±212.65	586.43±220.02	207.24±128.24	713.53±243.52*

[1]Data compared with unpaired t-test and statistically significant changes are indicated as ***p<0.001, ** p<0.01, *p<0.05.
